# Myeloid cell infiltration in skeletal muscle after combined hindlimb unloading and radiation exposure in mice

**DOI:** 10.1038/s41526-023-00289-w

**Published:** 2023-06-07

**Authors:** Eric B. Emanuelsson, Bjorn Baselet, Mieke Neefs, Sarah Baatout, Brit Proesmans, Lisa Daenen, Carl Johan Sundberg, Helene Rundqvist, Rodrigo Fernandez-Gonzalo

**Affiliations:** 1 Department of Physiology and Pharmacology, Karolinska Institutet, Stockholm, Sweden; 2 Radiobiology unit, SCK CEN, Belgian Nuclear Research Centre, Mol, Belgium; 3 Department of Learning, Informatics, Management and Ethic, Karolinska Institutet, Stockholm, Sweden; 4 Department of Laboratory Medicine, Division of Clinical Physiology, Karolinska Institutet, Stockholm, Sweden; 5 Unit of Clinical Physiology, Karolinska University Hospital, Stockholm, Sweden

**Keywords:** Physiology, Preclinical research

## Abstract

The skeletal muscle and the immune system are heavily affected by the space environment. The crosstalk between these organs, although established, is not fully understood. This study determined the nature of immune cell changes in the murine skeletal muscle following (hindlimb) unloading combined with an acute session of irradiation (HLUR). Our findings show that 14 days of HLUR induces a significant increase of myeloid immune cell infiltration in skeletal muscle.

Spaceflight presents multiple challenges to the organism, which must try to adapt to environmental stressors, primarily microgravity and ionizing radiation^1^. Radiation during missions in low Earth orbit (e.g., on the International Space Station) affects the health of astronauts^2^, and these effects will become even greater and more dangerous during missions in deep space due to the nature and dose of radiation^3,4^. Two of the most affected organs during space travel are the skeletal muscle and the immune system^5,6^. As an example, skeletal muscle of mice subjected to microgravity and radiation by means of hindlimb unloading (HLU; a validated spaceflight animal analog^7^) display a rapid loss of muscle mass and show dysregulation of critical molecular regulators of muscle growth and metabolism^8^. Interestingly, there is growing evidence of strong associations between growth, metabolism, and inflammation in skeletal muscle^9,10^. It follows that alterations in the immune system due to space stressors might in turn modify the molecular underpinnings within the muscle, potentially exacerbating the deleterious effects of microgravity and radiation on this organ. However, the mechanisms explaining the crosstalk between skeletal muscle and the immune system under space-like conditions are far from being understood. During regenerative muscle processes under normal gravity and ionizing radiation conditions, such crosstalk appears to be mediated by infiltration of myeloid and lymphoid immune cells into the muscle^11–13^. It is unknown, however, whether the space environment triggers any response in the myeloid and/or lymphoid immune cell populations present in the skeletal muscle.

In view of the lack of information regarding the consequences of spaceflight on the immune system-skeletal muscle crosstalk and integrity, we designed a study to investigate potential immune-cell alterations occurring within the skeletal muscle after a period of unloading and radiation (HLUR) exposure in mice. We focused our efforts in determining the nature of immune cell changes in the skeletal muscle from a gene expression perspective, to then confirm our findings using an immunohistochemistry approach. From the above, we hypothesized that HLUR would induce an infiltration of myeloid and lymphoid cells in the skeletal muscle.

Following 14 days of hindlimb unloading and an acute session of radiation (day 7), a significant decrease in total wet *M. soleus* but not *M. gastrocnemius* weight was found. (Fig. 1a, b). The reduction in *M. soleus* weight co-occurred with an increased gene expression of myostatin, a well-known inhibitor of muscle mass^9^, which was not found in *M. gastrocnemius* (Fig. 1c, d). Myostatin induces muscle atrophy by up-regulating atrophy-related genes and by inhibiting the Akt/mTOR signaling pathway^9^. The Akt/mTOR signaling pathway was recently found to be dysregulated following unloading and irradiation^8^. Additionally, the same pathway has been suggested to be down-regulated following increased levels of inhibitory cytokines, such as TNF-α^9^, indicating a potential immune-skeletal muscle crosstalk under atrophy stimulating conditions.

**Fig. 1:**
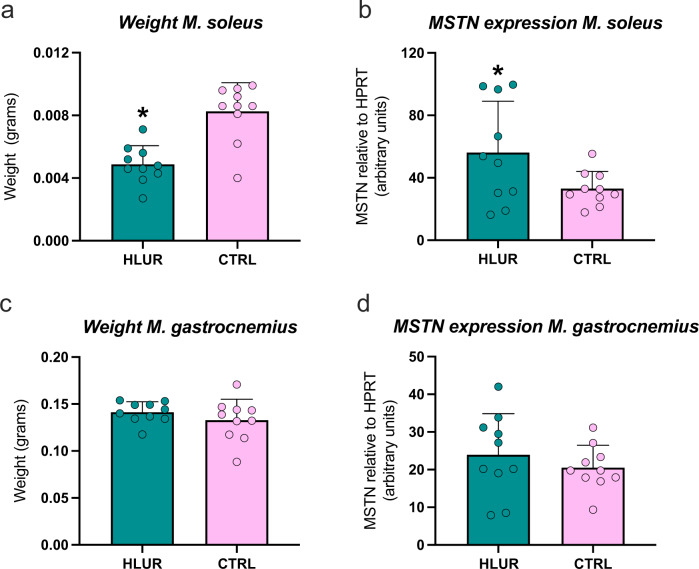
Weight and myostatin (MSTN) gene expression levels after hindlimb unloading and radiation. **a**, **b** weight and MSTN levels of M. soleus. **c**, **d** weight and MSTN levels of M. gastrocnemius. HLUR hindlimb unloading and radiation group, CTRL control group. *Significantly different than CTRL group (unpaired *t*-tests; *p* < 0.05). Error bars represent standard deviation.

To understand the mechanisms behind this crosstalk following HLUR, gene expression analyses of known immune markers were performed in *M. soleus*. HLUR increased the expression of the myeloid marker CD11b (*p* = 0.05) and a tendency towards increased pro-inflammatory cytokine TNF-α (*p* = 0.07) was observed, while no differences were found between HLUR and CTRL in lymphoid markers such as CD4, CD8, and CD20. In addition, no difference in INF-γ or IL-6 levels were found (Fig. 2a). To get a better understanding of the myeloid immune cell types driving these increased inflammatory markers, additional markers for the immune cells present in skeletal muscle tissue were analyzed. A significant increased expression (*p* < 0.05) of ICAM-1, H2-Ab, CD11c, CD86 and MMP-12 was found, supporting the induction of myeloid but not lymphoid cell infiltration after HLUR (Fig. 2b, c). The increased gene expression of these inflammatory markers could reflect a compensatory mechanism for the ongoing atrophy and/or a radiation-induced immune response. Indeed, past research has shown impaired healing processes following skeletal muscle injury in macrophage depleted mice^14^.

**Fig. 2:**
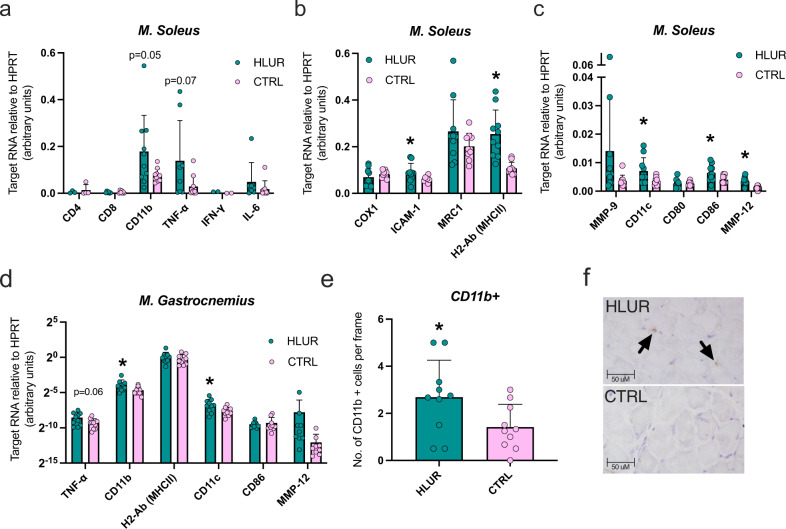
Myeloid, but not lymphoid, immune cell infiltration after hindlimb unloading and radiation. **a**–**c** gene expression of inflammatory, myeloid immune cells, and lymphoid immune cells markers in M. soleus. **d** gene expression of inflammatory, myeloid immune cells, and lymphoid immune cells markers in M. gastrocnemius (*X* axis in log ^-2 scale). **e** number of CD11b positive cells in M. gastrocnemius. **f** example of cross sections used to detect CD11b positive cells. HLUR hindlimb unloading and radiation group, CTRL control group. *Significantly different than CTRL group (unpaired *t*-tests; *p* < 0.05). Error bars represent standard deviation.

As a next step, we assessed if the increments of inflammatory markers following HLUR occurred also in the *M. gastrocnemius*. A significant increase of CD11c (*p* = 0.02) and CD11b (*p* = 0.048), a trend towards an increased expression of TNF-α (*p* = 0.06) was found, while H2-Ab and CD86 were unchanged (Fig. 2d). Thus, similar to the results found for *M. soleus*, HLUR seemed to provoke an induction of myeloid cells in the *M. gastrocnemius*. These findings, especially those in *M. gastrocnemius*, suggest that increased inflammation precedes muscle atrophy. This hypothesis is in line with previous work showing an enrichment of KEGG pathways and gene ontology terms related to increased inflammatory response before muscle atrophy in unloaded rats^15^.

To validate the increased gene expression of inflammatory markers following HLUR, immunohistochemical staining of CD11b was performed. In line with the increased gene expression of CD11b, a significantly higher number of CD11b positive cells per area were found in skeletal muscle from HLUR compared with CTRL (*P* = 0.043; Fig. 2e, f). This result confirms that there is a myeloid cell infiltration in the skeletal muscle following combined radiation and unloading, which could be related to repairing mechanisms attempting to reduce muscle atrophy^16^. However, additional studies are needed to determine the specificity of particular immune cell population involvement in the immune-skeletal muscle crosstalk following spaceflight conditions. In addition, the use of radiation protocols more similar to cosmic rays and solar particle events than the X-rays used here should be considered to extrapolate the current results to real space conditions.

In summary, our findings show that 14 days of unloading and a session of radiation induces a significant increase of inflammatory and immune-cell markers in skeletal muscle. This may occur as a compensatory mechanism to delay the skeletal muscle atrophy. Importantly, the combined unloading and radiation protocol provoked an infiltration of myeloid, but not lymphoid, immune cells into the skeletal muscle tissue. These findings highlight the crosstalk between the skeletal muscle and the immune system during spaceflight conditions. Future studies are needed to improve our understanding of the dynamics and the specificity of immune cells involved in the immune-skeletal muscle crosstalk. Studies using single-cell technology together with omics approaches seem a particularly interesting strategy to advance the knowledge about the muscle-immune crosstalk during spaceflight.

## Methods

### Experimental design

Ten mice were subjected to 14 days of hindlimb unloading (HLU) by tail suspension with an acute radiation session (dose = 25 mGy, X-ray) on day 7 of unloading (HLUR group). Ten mice were used as control (similar cages, sham radiation). Mice were sacrificed and *Mm. soleus* and *gastrocnemius* were obtained. Muscle samples were used to analyze gene markers for inflammation and specific immune cells. Finally, muscle samples underwent immunohistochemistry analysis to confirm the findings found at a gene expression level. The methods were performed in accordance with international (European Union) and national (Belgium) guidelines and regulations and approved by Medanex Clinic (EC MxCl 2018–100).

### Animals

Twenty adult (14 weeks), male C57/BL6J mice were randomized into a control group (CTRL; *n* = 10) and an unloading and radiation group (HLUR; *n* = 10). Mice were single housed and kept on a 12:12-h light-dark cycle. Mice were diet-paired, i.e., CTRL mice ate what the paired HLUR mouse ate the day before.

### Hindlimb unloading

Unloading (i.e., microgravity on the rear limbs) was achieved by the tail suspension technique. In brief, under sedation, the tail was attached to the suspension device of the cage at three points along its length. Once the mice were suspended in the device, their hind limbs were not allowed to touch the metal grid on the cage floor. The suspension angle between the animal and the floor averaged 30°. The unloading intervention was maintained for 14 days. After this period, (or 14 days in similar cages and conditions for CTRL animals) mice were sacrificed and *Mm. soleus* and *gastrocnemius* muscles from both rear limbs were dissected, weighed and frozen in liquid nitrogen pre-cooled isopentane, and then kept at −80 °C until further analysis.

### Whole body irradiation during unloading

A total X-radiation dose of 25 mGy was delivered (HLUR mice). This dose is comparable to the total amount of effective dose astronauts encounter during a 2-week deep space mission^17^. During irradiation mice were kept inside their respective cages wrapped in breathable autoclave bags to maintain the specific-pathogen-free conditions. CTRL mice were sham-irradiated, i.e., bagging, transferred in cages by car to radiation facility, and then returned to the animal facility to experience the same thermal conditions and potential transportation-induced stress as HLUR.

### Gene expression analysis

One of the *M. soleus* and ~20 mg of one of the *M. gastrocnemius* from the rear limbs from all mice were homogenized in TRIzol and total RNA was extracted and reverse transcribed to cDNA. Gene-expression (mRNA) was determined using quantitative real-time PCR procedures. Gene-specific primers for markers of immune/inflammatory processes were used. The transcripts panel tested was the following: CD4, CD8, CD20, IFN-γ, CD11b, CD11c, MHCII, TNF-α, IL-6, CD206, iNOS, Arginase, CD86, CD80, PTGS, iCam, and HPRT (control/reference gene).

### Immunohistochemical analysis

One of the *M. gastrocnemii* from all mice was oriented for transverse sectioning and 5 μm cross-sections were cut in a cryostat at −22 °C and mounted on glass slides. Sections were stained using monoclonal antibodies for Cd11b (BioLegend, rat IgG2b, clone M1/70) to analyze myeloid cell infiltration. The stained cross sections were then captured using bright field microscopy with 20x magnification (Leica DMLA, Leica application suite X software). Three to four pictures representing a fixed area of 1.96 mm^2^ per picture were captured in each cross-section (average cross sections per sample; 3.5). A semi-automatic pipeline used to detect Cd11b specific staining was employed using ImageJ software for Mac OS X. The number of positive cells was then averaged across all cross-sections.

### Data analysis

Unpaired *t*-tests were used to compare muscle weight, gene expression, and cell infiltration, between the HLUR and CTRL groups. The level of significance was set at 5% (*p* < 0.05). All statistical analyses were performed using Prism 9 for Mac OS X (GraphPad Software, San Diego, CA).

### Reporting summary

Further information on research design is available in the Nature Research Reporting Summary linked to this article.

## Supplementary information

Reporting Summary

## Data Availability

The data supporting the conclusions of this article will be made available by the authors upon reasonable request.
