# Supplementary material for: Myeloid cell infiltration in skeletal muscle after combined hindlimb unloading and radiation exposure in mice
Source: NPJ Microgravity. 2023 Jun 7;9:40. doi: 10.1038/s41526-023-00289-w (PMC10247796; doi:10.1038/s41526-023-00289-w)
Supplement: Supplementary file 1 — Reporting Summary [file 41526_2023_289_MOESM1_ESM.pdf]

nature portfolio

Corresponding author(s): Rodrigo Fernandez-Gonzalo
Last updated by author(s): Jan 20, 2023

Reporting Summary

Nature Portfolio wishes to improve the reproducibility of the work that we publish. This form provides structure for consistency and transparency in reporting. For further information on Nature Portfolio policies, see our Editorial Policies and the Editorial Policy Checklist.

Statistics

For all statistical analyses, confirm that the following items are present in the figure legend, table legend, main text, or Methods section.

n/a	Confirmed	
☐	☒	The exact sample size (*n*) for each experimental group/condition, given as a discrete number and unit of measurement
☐	☒	A statement on whether measurements were taken from distinct samples or whether the same sample was measured repeatedly
☐	☒	The statistical test(s) used AND whether they are one- or two-sided<br>*Only common tests should be described solely by name; describe more complex techniques in the Methods section.*
☒	☐	A description of all covariates tested
☒	☐	A description of any assumptions or corrections, such as tests of normality and adjustment for multiple comparisons
☐	☒	A full description of the statistical parameters including central tendency (e.g. means) or other basic estimates (e.g. regression coefficient) AND variation (e.g. standard deviation) or associated estimates of uncertainty (e.g. confidence intervals)
☒	☐	For null hypothesis testing, the test statistic (e.g. *F*, *t*, *r*) with confidence intervals, effect sizes, degrees of freedom and *P* value noted<br>*Give P values as exact values whenever suitable.*
☒	☐	For Bayesian analysis, information on the choice of priors and Markov chain Monte Carlo settings
☒	☐	For hierarchical and complex designs, identification of the appropriate level for tests and full reporting of outcomes
☒	☐	Estimates of effect sizes (e.g. Cohen's *d*, Pearson's *r*), indicating how they were calculated

Our web collection on statistics for biologists contains articles on many of the points above.

Software and code

Policy information about availability of computer code

Data collection	A semi-automatic pipeline used to detect Cd11b specific staining was employed using ImageJ software for Mac OS X
Data analysis	Data was analyzed using Prism 9 for Mac OS X (GraphPad Sofware, Sand Diego, CA)

For manuscripts utilizing custom algorithms or software that are central to the research but not yet described in published literature, software must be made available to editors and reviewers. We strongly encourage code deposition in a community repository (e.g. GitHub). See the Nature Portfolio guidelines for submitting code & software for further information.

Data

Policy information about availability of data

All manuscripts must include a data availability statement. This statement should provide the following information, where applicable:

- Accession codes, unique identifiers, or web links for publicly available datasets
- A description of any restrictions on data availability
- For clinical datasets or third party data, please ensure that the statement adheres to our policy

The data supporting the conclusions of this article will be made available by the authors upon reasonable request.

Human research participants

Policy information about studies involving human research participants and Sex and Gender in Research.

Reporting on sex and gender	*Use the terms sex (biological attribute) and gender (shaped by social and cultural circumstances) carefully in order to avoid confusing both terms. Indicate if findings apply to only one sex or gender; describe whether sex and gender were considered in study design whether sex and/or gender was determined based on self-reporting or assigned and methods used. Provide in the source data disaggregated sex and gender data where this information has been collected, and consent has been obtained for sharing of individual-level data; provide overall numbers in this Reporting Summary. Please state if this information has not been collected. Report sex- and gender-based analyses where performed, justify reasons for lack of sex- and gender-based analysis.*
Population characteristics	*Describe the covariate-relevant population characteristics of the human research participants (e.g. age, genotypic information, past and current diagnosis and treatment categories). If you filled out the behavioural & social sciences study design questions and have nothing to add here, write "See above."*
Recruitment	*Describe how participants were recruited. Outline any potential self-selection bias or other biases that may be present and how these are likely to impact results.*
Ethics oversight	*Identify the organization(s) that approved the study protocol.*

Note that full information on the approval of the study protocol must also be provided in the manuscript.

Field-specific reporting

Please select the one below that is the best fit for your research. If you are not sure, read the appropriate sections before making your selection.

☒ Life sciences ☐ Behavioural & social sciences ☐ Ecological, evolutionary & environmental sciences

For a reference copy of the document with all sections, see nature.com/documents/nr-reporting-summary-flat.pdf

Life sciences study design

All studies must disclose on these points even when the disclosure is negative.

Sample size	The null hypothesis and the alternative hypothesis that will be tested based on an important outcome/variable of the study and this variable should be clearly described:<br>• Hypotheses<br>o Clinical variable: Lymphocyte count in whole blood after exposure to the combined space<br>o Null hypothesis: No change in lymphocyte count in whole blood after exposure to the combined space stress<br>o Alternative hypothesis: Decreased lymphocyte count in whole blood after exposure to the combined space stress<br>• The model/test is used to test this hypothesis: paired T-test<br>o The minimum significant deviation from the null hypothesis to be expected: We wish to obtain a minimal significant deviation from the null hypothesis of 0.66 or 66%<br>o The probability with the minimum significant deviation from the null hypothesis is intended to be called power: we want a power of 0.80 of 80%<br>o The maximum probability of a false discovery, i.e. the significance of the test, i.e. the type I error rate: We want a type I error rate of 0.05 or 5%<br>With the aforementioned input variables, we calculated the required sample size with G*Power version 3.1.9.7. Based on these input parameters we would require a sample size of 10.
Data exclusions	n/a
Replication	n/a
Randomization	Pure randomization of mice to control or HLUR group
Blinding	Blinding for IHC analysis was performed by coding all the images by one researchers. Then, other member of the group blinded to the coding performed the analyses

Reporting for specific materials, systems and methods

We require information from authors about some types of materials, experimental systems and methods used in many studies. Here, indicate whether each material, system or method listed is relevant to your study. If you are not sure if a list item applies to your research, read the appropriate section before selecting a response.

Materials & experimental systems

n/a	Involved in the study	
☒	☐	Antibodies
☒	☐	Eukaryotic cell lines
☒	☐	Palaeontology and archaeology
☐	☒	Animals and other organisms
☒	☐	Clinical data
☒	☐	Dual use research of concern

Methods

n/a	Involved in the study	
☒	☐	ChIP-seq
☒	☐	Flow cytometry
☒	☐	MRI-based neuroimaging

Animals and other research organisms

Policy information about studies involving animals; ARRIVE guidelines recommended for reporting animal research, and Sex and Gender in Research

Laboratory animals	C57/BL6J mice
Wild animals	n/a
Reporting on sex	male
Field-collected samples	Mice were single housed and kept on a 12:12-hr light-dark cycle. Room temperature was kept constant.
Ethics oversight	The methods were performed in accordance with international (European Union) and national (Belgium) guidelines and regulations and approved by Medanex Clinic (EC MxCl 2018-100).

Note that full information on the approval of the study protocol must also be provided in the manuscript.
